# Towards a Fungal Science That Is Independent of Researchers’ Gender

**DOI:** 10.3390/jof8070675

**Published:** 2022-06-28

**Authors:** Nada Kraševec

**Affiliations:** Department of Molecular Biology and Nanobiotechnology, National Institute of Chemistry, SI-1000 Ljubljana, Slovenia; nada.krasevec@ki.si

**Keywords:** female researchers, fungal genetics conference, gender disparity, inclusion of sex and gender in research, mycology journals, pathogenic fungi, unconscious bias, women in science

## Abstract

The main drivers of gender mainstreaming in basic and clinical research appear to be funding agencies and scientific journals. Some funding agencies have already recognized the importance of their actions for the global development of ideas in science, but further targeted efforts are needed. The challenges for women scientists in fungal research appear to be similar to those in other science, technology, engineering, and mathematics disciplines, although the gender gap in mycology publishing appears to be less pronounced; however, women are underrepresented as last (corresponding) authors. Two examples of best practices to bridge the gap have been promoted in the fungal community: “power hour” and a central resource database for women researchers of fungi and oomycetes. A more balanced ratio of women researchers among (plenary) session speakers, (plenary) session chairs, and committee members at the recent fungal genetics conference is an encouraging sign that the gender gap can be closed. The editorial policy of some journals follows the guidance “Sex and Gender Equality in Research,” and other journals should follow, and indicate the gender ratio among authors and reviewers.

## 1. Introduction

Fungi have tremendous positive and negative impacts on our daily lives [1,2]. From more than 1.5 million species (or even up to 6 million species), only about 5% have been described despite all the efforts of fungal researchers. Fungi first appeared as early as 1.5 billion years ago and were among the first organisms domesticated by humans [3]. They serve us as industrial biofactories, although the enormous possibilities and opportunities offered by their compounds remain unexplored. Fungal biotechnology may help human society transition from a petroleum-based to a bio-based circular economy. Fungi are capable of sustainably producing food, feed, chemicals, fuels, textiles, and materials for construction, transportation, furniture, and more [1,2]. In addition, fungi also produce toxins that spoil our food and they primarily infect immunocompromised patients, driving up pest control and public health costs [1,2]. However, only about 100 to 200 of all fungal species have developed successful strategies to parasitize human hosts [3,4,5,6]. Understanding the occurrence of pathogenic fungi is particularly important for predicting the potential emergence of new human pathogens that could result from climate change.

Society is slowly becoming aware that sex/gender plays an important role not only in our daily lives, but also in scientific research. It was not so long ago that scientific research was conducted almost exclusively by men and for men, and the technical products developed were tailored to men or users, according to the traditional social roles of men and women [7]. Some funding agencies, which seem to be one of the main drivers of research, have already recognized the importance of considering sex and gender in basic and clinical research [8]. Understanding sex and gender in health research can improve the quality of funding and health outcomes. Funding agencies and scientific journals are two checkpoints for knowledge acquisition and dissemination, including consideration of sex/gender in research. A survey of 45 national funding agencies in Europe, the United States and Australia found that (i) mention of sex/gender in funding and publication policies is not uniform; (ii) there is wide variation in how sex/gender is conceptualized, and how researchers are required to consider sex/gender in research; (iii) funding agencies tend to prioritize gender equality in research groups and funding over sex/gender mainstreaming in research content and knowledge production; (iv) with few exceptions, agency and journal criteria do not recognize the complexity of the sex/gender dimension, including the intersection of sex/gender with other important health determinants [9].

Sex refers to a set of biological attributes in humans and animals that are associated with physical and physiological features, including chromosomes, gene expression, hormone function, and reproductive/sexual anatomy [10]. Sex is usually categorized as female or male, although there is variation in the biological attributes that constitute sex and how those attributes are expressed. Gender refers to the socially constructed roles, behaviors, and identities of female, male, and gender-diverse people [10]. Gender is usually incorrectly conceptualized as a binary (female/male) factor. In reality, there is a spectrum of gender identities and expressions defining how individuals identify themselves and express their gender [11].

Gender equality is one of the fundamental values of the European Union. Since 2012, gender equality in research and innovation has been progressively strengthened as a priority, and the proposed actions focus on three main areas: (i) promoting gender equality in careers, (ii) ensuring gender balance in decision-making processes, and (iii) integrating the gender dimension in research and innovation content and programs [12]. The aim of sex and gender analysis is to promote rigorous, replicable, and responsible science [13]. Incorporating sex and gender variables into experimental design has already enabled advances in a number of disciplines: in major chronic diseases, targeted human therapeutics, climate impacts in the ocean, designing safer products, or reducing gender bias in artificial intelligence [14,15]. A provided roadmap for sex and gender analysis across scientific disciplines calls on researchers, funding agencies, peer-reviewed journals, and universities to coordinate the implementation of robust methods for sex and gender analysis [14].

A numerical assessment of gender gaps can identify areas where equality cannot be achieved without intervention, reveal undesirable biases, and provide criteria for gender balance in conference speakers, editors, and employment committees [16]. Gender parity refers to a ratio of 50:50 between women and men; gender balance means that the proportion of women and men is between 40% and 60% of the total population; underrepresentation and overrepresentation means the proportion of women or men is below 40% or above 60%, respectively [13]. However, less than 2% of publications included a gender dimension in their research content; this percentage has increased by less than 1% since 2010 [13]. Publications in medical and health sciences were the most likely to include a gender dimension, while publications in engineering and technology were the least likely to include such a dimension. At the country level, less than 10% of Horizon 2020 projects from the EU’s research and innovation funding program in the period from 2014 to 2020, contained a gender dimension [13]. Editorials often express the opinions and new decisions of journal editors. The number of published editorials on sex/gender was low until 1990, then increased rapidly, and then steadily from 1994 to 2013. Since 2014, we can observe the interest of editors in this topic, as they publish about 200 editorials on sex/gender every year [17].

Therefore, it is essential for scientific journals to follow the Sex and Gender Equality in Research guidelines [11]. These guidelines emphasize the need to differentiate subjects by sex/gender, analyze results by sex/gender, and identify meaningful differences whenever possible, even if they were not initially expected [17]. The guidelines include a series of questions to help authors prepare and review their papers to determine whether they have adequately addressed sex/gender-specific issues [11,18]. The guidelines encourage editors to recommend that authors improve their sex and gender presentation before peer review. The guidelines also help peer reviewers to check for compliance with the above guidelines during the process [11,17].

Two different forums of researchers not originally involved in sex and gender research have recently discussed the dimensions of sex and gender in scientific research: the Plotina project and the annual event of the Slovenian Commission for Equal Opportunities in Science [7,19]. The aim of the H2020 Plotina project was to create conditions in which research organizations are able to develop, implement, and evaluate their gender-equality plans that contribute to such changes in the working environment that women and men have equal opportunities to realize their potential, goals, and needs in research and science. The final conference of the project, entitled “ReGendering Science—For an inclusive research environment” was held at University Alma Mater Studiorum in Bologna [19]. The traditional conference of the Commission for Equal Opportunities in Science, the advisory body of the Slovenian Ministry of Education, Science and Sport, was held at the Research Center of the Slovenian Academy of Sciences and Arts in Ljubljana on the topic “The Overlooked Dimensions of Gender in Scientific Research” [7]. Researchers presented their experiences of integrating the gender dimension in their research and described the thinking process, possible doubts or surprises to investigate to what extent the gender dimension is already taken into account in research today and where we still overlook it [19].

As a result of these two events, a paper has been published that considers sex/gender/mating type in the study of fungal infections in humans, highlighting some aspects that may be overlooked in the overall picture [20]. Sex and age of participants in antifungal trials are critical to interpret results, but researchers should already in preclinical studies report the sex of model (primary) cells or cell-culture models, and model animals [15,21,22,23]. Awareness of early detection and treatment of fungal infections also depends on gender [24]; the general population needs to be better educated about fungal diseases. The sex of the human host affects the incidence of some fungal infections because the human immune response to fungal infections is also sex-dependent [25,26,27,28,29]; further studies are needed. The mating types of some fungal species appear to be associated with some human infections [3,30,31,32,33]. Sexual and parasexual reproduction of some fungi is an important mechanism for the development of resistance to antifungal drugs [34]. Globalization and climate change will exacerbate the already existing challenges of fungal infections in humans; we need more multidisciplinary approaches to address them [20].

This paper discusses the gender imbalance in research participation, scientific career advancement, and scientific publishing. The author analyzes the gender gap in scientific publishing in mycology and presents best practices to mitigate the challenges faced by female fungal researchers. The first example is the introduction of a so-called “power hour” into the scientific conference program; the second example is a central resource database to identify potential women speakers and representatives on nominating committees at scientific meetings in the field. The results of actions to overcome the inequality gap are analyzed as contributions from speakers, chairs, and committee members at fungal genetics conferences.

## 2. Materials and Methods

### 2.1. Visualizing of the Gender Differences in Mycology Publications

The author analyzed published data that were accessible through the web application The Gender Gap in Academic Publishing [35]. These data were collected by downloading all ~27 million records on PubMed (2000–2016), and the gender of authors was determined by matching their first names to the genderize.io database [16,35]. This web application catalogs selected journals into multiple disciplines [35], which were assigned to a research discipline using PubMed’s categorization scheme. In the visualization, data from the last year’s survey (2016) were highlighted in numbers, and the future gender ratio of authors of scientific publications was estimated for the current year (2022). Since the discipline of mycology was not available in this web application, the author selected journals from the category mycology, according to the Journal Citation Reports (JCR) database published by Clarivate Analytics for 2016 (Table S1) [36]. Most of these thirty journals from the mycology category belonged to the discipline of microbiology. These journals were FEMS Yeast Research, Fungal Biology, Medical Mycology, Mycologia, Mycopathologia, Mycoses, Revista Iberoamericana de Micologia, and Yeast. Other journals in the mycology category have been assigned to botany—Mycorrhiza; cell biology—Eukaryotic Cell; or genetics—Fungal Genetics and Biology. For clarity, data accessible through this web application are limited to combinations for which a sufficiently large sample size is available in terms of number of publications (at least on hundred), years (at least five), and authors (at least fifty per year for five or more years). Several journals from in the JCR mycology category, such as Cryptogamie, Mycologie, Fungal Biology Reviews, Fungal Diversity, Fungal Ecology, IMA Fungus, International Journal of Medicinal Mushrooms, Journal de Mycologie Medicale, Lichenologist, Mycobiology, Mycokeys, Mycological Progress, Mycoscience, Mycosphere, Mycotaxon, Mycotoxin Research, Persoonia, Studies in Mycology, Sydowia, and World Mycotoxin Journal, did not meet the listed criteria and were therefore excluded from the comparison. Other mycological journals may not have begun publishing scientific articles until after the year 2016, such as the Journal of Fungi. The journal Mycological Research was discontinued in 2011. As fungal research is often published by multiple journals from other disciplines, multidisciplinary journals Science and Nature were chosen as a comparison. The gender ratio was estimated as the percentage of women authors.

### 2.2. Analysis of the Comprehensive Table Compiled by Women Researchers in Fungi & Oomycetes

The comprehensive table has been compiled since 2016 by Women Researchers in Fungi & Oomycetes [37] on a voluntarily basis. Data from 300 women researchers from around the world were analyzed through 17 January 2020 (this number was about 10% higher in May 2022). The corresponding career stages of the women researchers were reported as junior researcher, which means assistant professor or similar position (grade C); mid-level position (grade B); and full-time research position, that is, a professor or equivalent (grade A). These data were presented as percentages of all career stages entered. The geographic location of the job was divided into Africa, Asia, Australia and New Zealand, Europe, and North and South America; these data were presented as percentages of all geographic locations entered. To describe the research area, researchers selected two options from several assigned subfields: biochemistry (a), biotechnology (b), cell biology (c), development (d), ecology (e), evolution (f), genetics or genomics (g), immunology (h), medical mycology (i), plant pathology (j), signaling (k), and taxonomy (l); these data were presented as percentages of all entered subfields. The author used the Tagul Word Cloud Art Creator web tool [38] to analyze freely chosen keywords. These keywords were evaluated as single words; common words were omitted, resulting in 400 keywords. For example, the most common term plant occurred in sixteen different combinations: fungal-plant-interactions, fungal-plant-pathogens, fungal-plant-symbiosis, oomycetes-plant-interaction, plant-biomass-degradation, plant-defense, plant-disease, plant-pathologist, plant-fungal-ecology, plant-fungal-interactions, plant-microbe-interactions, plant-microbial-interactions, plant-pathology, plant-resistant-to-fungal-disease, plant-resistant-to-fungal-pathology, and plant-soil-microbe-interaction. From the keywords repeated at list twice, a world cloud was drawn.

### 2.3. Analysis of the Gender of Speakers, Session Chairs, and Committee Members

To assess progress in narrowing the gender gap, the author selected two conferences organized prior to the year 2016 (prior compilation of the central resource table and introduction of power hours to the meetings): the 28th Fungal Genetics Conference in Asilomar, 17–22 March 2015 (FGC28), and the 13th European Conference on Fungal Genetics in Paris, France, 3–6 April 2016 (ECFG13). The two most recent conferences of the same type held after the year 2019 served for comparison: the 15th European Conference on Fungal Genetics Rome, Italy, 17–20 February 2020 (ECFG15) and the 31st Fungal Genetics Conference in Asilomar, 15–20 March 2022 (FGC31). The format of the FGC31 was hybrid, including online speakers and participants. The number of women (plenary) speakers compared to the total number of (plenary) speakers, the number of women (plenary) chairs compared to total number of (plenary) chairs, and the number of women members on committees compared to the total number of committee members were determined according to their announcement in the abstracts of program books [39,40,41,42]. In five cases, it was not possible for the author to determine the gender of the participants.

## 3. Results and Discussion

### 3.1. Is There a Gender Imbalance in Women’s Participation in Research and in Scientific Career Advancing?

To obtain clear background information for this study, the author first evaluated a comprehensive source of combined statistical data on gender ratios in research, produced by the European Commission and updated every three years [13]. At the European level, the proportion of women among doctoral graduates had almost reached gender parity in 2018 (48.1%) [13]. The most popular broad field of study for women doctoral graduates, health and welfare, accounted for 26.1% of all women (and 15.9% of all men), resulting in an overrepresented share of 60.3% of women among doctoral graduates in 2018. In the broad field of natural sciences, mathematics, and statistics, which was the most popular among men doctoral graduates (27.4% and 24.1% of women, respectively), this proportion was 44.9% [13].

Among European researchers, the proportion of women was 32.8% in 2018; in particular, 43.9% in the government sector, 42.3% in the higher education sector, and only 20.9% in the business sector [13]. There were significant differences by grade: While women made up, on average, almost a parity of the academic staff in grades C and D (46.6% and 47.1%, respectively) and 40.3% of the staff in grade B, they occupied only about 26.2% of positions in grade A [13]. Grade B researchers work in positions that are not as senior as the top position, but are higher than newly qualified doctoral graduates (Grade C); grade D are researchers without PhDs. Grade A corresponds to a full professorship in most countries and is the highest grade at which research is normally conducted within the institutional or corporate system. Women were underrepresented among grade A academic staff in all research and development fields, with the lowest proportion in the natural sciences (22%) (along with engineering and technology) [13]. Although there have been some slight improvements, women have had greater difficulty than men in advancing to top academic positions. Women were most underrepresented among grade A staff who were 55 or older, the age group in which the proportion of grade A staff was the highest for both women and men [13]. The proportion of women among the heads of universities or equivalent institutions conducting doctoral studies has improved over time, but several countries still lag behind. At the European and national levels, women have been underrepresented among board members and heads [13]. The funding success rate is calculated as the number of research grantees relative to the number of applicants; the success rate of women is compared to the success rate of men. In 2019, women had a lower success rate than men in accessing funding at the European level in all research and development fields except agricultural sciences, and humanities and arts. Women researchers were least successful in applying for research funding in the natural sciences [13].

The glass ceiling is a phenomenon in which structural barriers impede women’s access to the highest decision-making and leadership positions in organizations. The glass ceiling index represents the relationship between the proportion of women in academia (Grades A, B, and C) and the proportion of women in top academic positions (Grade A) and provides a way to measure the extent of potential disadvantage for women specifically in the research community. It indicates how great the chances are for women to advance in their academic careers. The higher the value, the greater the impact of the glass ceiling and the more difficult it is for women to reach a higher position. At the European level, the glass-ceiling index score was about 1.5 in 2018 [13].

Mycology is a branch of biology that deals with the study of fungi, including their genetic and biochemical properties, their taxonomy and utility to humans, and their hazards, such as toxicity or infection. The exact proportion of women researchers in mycology can be estimated indirectly [13,43,44,45]. Most of the fungal research can probably be classified as natural sciences, mathematics, and statistics if research and development is broadly defined, and as biological and related sciences or the environment if more narrowly defined; however, some of the fungal research also fits into agriculture, forestry, fisheries, and veterinary, as well as health and welfare. As mentioned earlier, the proportion of women among doctoral graduates in 2018 by broad discipline was 44.9% for natural sciences, mathematics, and statistics; however, in the narrower field of natural sciences, the proportion of women among doctoral graduates in biological and related sciences was much higher, at 59.7% in 2018; in the narrower field of environment sciences, it was 56%; in agriculture, forestry, fisheries, and veterinary, it was 56.8%; and in health and welfare, it was 60.3% [13]. Using a database of thirty million profiles, career expert Zippia estimated the demographic and statistical data for microbiologists in the United States for 2019 and matched it with data from the U.S. Bureau of Labor Statistics, the U.S. Census Bureau, and current job openings. There were more than 20,000 microbiologists employed in the United States. An overrepresentative 60.8% of all microbiologists were women and 39.2% were men [28]. The average age of an employed microbiologist was 41 years, and the most common ethnicity of microbiologists was white (73.8%), followed by Asian (14.8%), and Hispanic or Latino (6.0%) [28].

Although it was directly estimated, women do seem to be almost overrepresented in fungal research.

### 3.2. Is There a Gender Imbalance in Scientific Publications in the Field of Fungal Research?

Although significant progress has been made in increasing women’s participation in research over the past decade, disparities persist, including in the authorship of research publications [46]. Combined statistical data on gender ratios in research suggested that there were more men than women among active authors at all seniority levels [13]. Active authors are defined as those who have authored ten or more papers in the last twenty years and at least one paper in the last five years, or those who have authored four or more papers in the last five years. Women were least represented as active authors in the natural sciences (and engineering and technology) and most represented in the medical and health sciences, and agricultural and veterinary sciences [13]. Seniority is estimated by the time elapsed since an author’s first publication in a journal: early-stage authors, whose first paper was less than five years ago; mid-stage authors, whose first paper was five to ten years ago; and senior authors, whose first paper was more than ten years ago. The gender gap was generally smaller for early-stage authors. As authors became more senior, women published increasingly fewer publications compared to men, and the gap widened to twice as many men authors as women authors [13]. Women were underrepresented in authorship teams, especially in international authorship teams, as shown by the ratio of women to men and the average proportion of women among authors. This is true across all research and development fields and follows trends in overall authorship, with the proportion being lowest in the natural sciences (as well as engineering and technology) [13]. Women were corresponding authors on half as many research publications as men, in addition, women were further underrepresented as corresponding authors in international collaborative publications [13]. The corresponding author, usually listed as last on the paper, is the person who has primary responsibility for communicating with the journal during the publication process and is responsible for several critical aspects of a study at each stage of its dissemination before and after publication. Usually a senior researcher or group leader is not only a major contributor to the paper, but is also in a position to ensure the smooth and successful publication process and to answer questions about the research after publication. However, women’s and men’s publications generally had equal citation impact according to the field-weighted citation impact, an indicator of a publication’s citation impact based on the actual number of citations for an article compared to the expected number of citations for articles of the same type, year of publication, and subject field [13].

In summary, women appeared as corresponding authors on half as many research publications as men. Combined with the finding that women, on average, formed less than half of the author teams and contributed to fewer publications than men, this suggests a complex situation in which women may have fewer opportunities than men to complete their research bibliography.

The gender of 36 million authors from more than a hundred countries who published in more than six thousand journals in most disciplines of science, technology, engineering, and mathematics was studied between 2000 and 2016, and a web application “The Gender Gap in Academic Publishing” was developed to provide easy access to the data and to predict future trends [16,35]. The difference in author positions in publications related to tenure is particularly striking, and prestigious journals have fewer female researchers as authors [16]. In addition, journals are twice as likely to invite men to submit papers [16]. Some rich countries had fewer female authors than poorer countries [16]. Despite recent progress, the gender gap is likely to persist for generations and will not be eliminated without further reforms in education, mentoring, and academic publishing [16].

**Figure 1:**
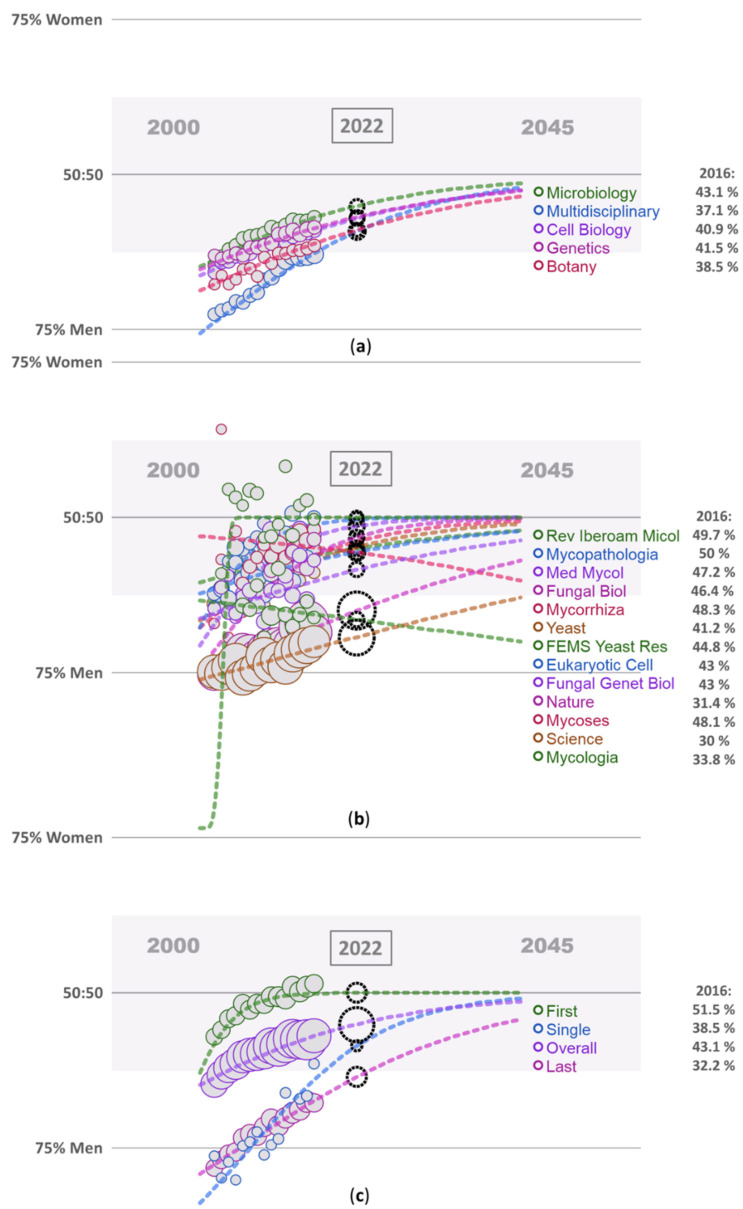
The gender gap in scientific publications. (**a**) Research disciplines covered by the mycological journals [36] (Tables S1 and S2); (**b**) selected journals covering mycology (Table S1); (**c**) Position in the author list in microbiology (Table S3). This visualization by the web app provides a view of the past, present, and estimated future (the abscissa, years) gender ratio of authors of scientific publications (the ordinate, percentages) [35]. The gender ratio was estimated by fitting a curve to the data [16]. Gender ratio of authors for 2016 (percentage of women researchers) is shown on the right; the black dashed circle shows the estimate for 2022, the size in corresponds to the number of papers. The overall position in the author list includes all authors.

The author used this web application to investigate the gender gap in mycology. Since the discipline of mycology was not directly accessible through the available web tool, the mycology discipline was analyzed using the journals listed in the JCR database in the mycology category for 2016 [35,36]. Only eleven journals from the mycology category were accessible through this web tool. For the others, the available sample size was not large enough in terms of number of papers, years, and authors. These journals were listed in four different disciplines, most in microbiology and one journal in genetics, cell biology, and botany. The percentage of women authors in microbiology fall within the range of gender balanced at 43.1%, and it is followed by genetics (41.5%), cell biology (40.9%), and was underrepresented in botany (38.5%) in 2016 (Figure 1a, Tables S1 and S2). The proportion of women authors in all listed disciplines was higher than in the multidisciplinary one (37.1%). All estimated trends for the listed disciplines indicate an increasing proportion of women authors in 2022 than recorded for 2016 (Table S2). A similar proportion of women authors as for microbiology was calculated out from mycological journals for mycology (45.1%) (Table S4).

In selected mycological journals, the proportion of women authors was, in Mycologia, 33.5%, followed by gender-balanced journals Yeast, Fungal Genetics and Biology, Eukaryotic Cell, and FEMS Yeast Research between 41.2% and 44.8% (Figure 1b). In some of listed mycological journals, this proportion was close to gender parity of authors: Fungal Biology, Medical Mycology, Mycoses, Mycorrhiza, and Revista Iberoamericana de Micologia between 46.4% and 50%, and gender parity was reached in Mycopathologia (Figure 1b). In contrast, in the two multidisciplinary established peer-reviewed journals Science and Nature, the proportion of women authors was less than one-third of overall authors (30% and 31.7%, respectively). Except for Mycoses and Mycologia, the estimated trend for listed journals in 2022 is positive (Science 30.7% and Nature 34.9%, respectively) (Figure 1b). The higher proportion of women authors within the listed mycological journals than in the comparison prestige journals was not surprising, but still informative about the dimension of the gender gap in fungal research.

However, when the numbers of women authors in microbiology or mycology (overall percentage 43.1% or 45.1%, respectively) were broken down by the author position on the paper, the gender gap becomes apparent even in microbiology or mycology (Figure 1c, Tables S3 and S4). Although female researchers were listed as first authors at (almost) a gender parity of 51.5% or 49.1%, respectively, only one-third of them were corresponding authors (in 2016, 32.2% or 33%, respectively), with 36.4% or 32.7%, respectively, predicted for the year 2022 (Figure 1c, Tables S3 and S4). According to estimates, male researchers were about twice more likely to be listed as last and usually corresponding authors who submit papers to these journals than female researchers were.

On the other hand, not all fungal research is published in mycological journals. For example, research on fungal aegerolysins (37 papers) can be attributed to twelve different disciplines, depending on the journals in which the papers were published [47,48,49,50,51]: most of which were attributed to microbiology, with eight papers (21%) (including three in mycology); but also to biochemistry, seven (19%); biophysics, three (8%); biotechnology, cell biology, and medicine, two (5%); and genetics, immunology, multidisciplinary, toxicology, and zoology, one (3%); and unsigned, eight papers (22%) (Table S5). The proportion of women authors was higher only in immunology than in microbiology, a balanced ratio was also achieved in genetics, toxicology, and cell biology; in medicine, biotechnology, biology, multidisciplinary, biochemistry, biophysics, and zoology (also with a negative trend for 2022), women were underrepresented as overall authors (Table S2).

In general, the gender gap in scientific publications is present in mycology, although it appears to be smaller than in other disciplines listed. However, as authors became more senior, the gap also widened in mycology to twice as many male authors as female authors, which is similar to the ratio in other research disciplines. The small number of mycological journals included is a limitation in estimating the gender gap in mycology. The new generation of young women researchers in mycology appears to be large enough; efforts should be made to break the glass ceiling and to retain senior women researchers in science. Scientific journals could help close the gender gap more quickly by openly reporting the gender ratio among authors (first, last, and overall) and reviewers involved in reviewing submitted manuscripts (just as some of them report the citation index) and including a statement of compliance with guidelines for the Sex and Gender Equality in Research, along with other statements at the end of the paper.

### 3.3. What Are the Challenges for Women in Fungal Research and Actions to Bridge the Gap That Were Identified in the So-Called “Power Hours”?

There are many reasons for the glass-ceiling effect: the persistence of gender stereotypes and prejudices about women’s abilities and roles in society, leading to direct and indirect discrimination during their careers [13,52]. A non-gender-sensitive work culture also includes a lack of arrangements that are compatible with family responsibilities; incidents of sexual harassment, bullying, and gender-based violence; and gender differences in individual decisions and behaviors are other barriers to career advancement [13]. At earlier stages in their careers, women leave research positions due to systemic biases, lack of advancement opportunities, challenges in work–life integration, imbalanced gender roles in household management, and care of children and the elderly. At a time when women are likely to assume leadership positions in their teams or fields, menopause also occurs [53]. Menopause is a natural life stage that is even more common than pregnancy or motherhood. The decline in reproductive hormones increases the risk for certain health problems; symptoms may include memory and concentration difficulties, which are important for research, teaching, and academic work. Many women working in science, technology, engineering, and mathematics are reluctant to talk about it for fear of being labeled as problematic [53]. Guidance tools already in place should be incorporated into gender equality plans, because for many, the years after menopause are among the most productive and rewarding when they have the opportunity to continue their scientific careers [53]. In addition, there is the gatekeeper phenomenon, where managers (often men) unconsciously promote the careers of people who are similar to them [12,13,54].

The U.S. Gordon Research Conferences, a conference center known for discussing cutting-edge research, is also an example of best practice. In addition to an excellent scientific program, the Gordon Research Conferences introduced the so-called “power hour” for the first time in 2016. This part of the conference provides an opportunity for all attendees to discuss the challenges of women in science (as well as issues of inclusion, diversity, and minorities). In 2017, the conference, entitled “Immunology of Fungal Infections,” included a “power hour” for the fungal community for the first time [55]. In 2018, it was included in the program of the conference, entitled “Molecular and Cellular Biology of Fungi,” and in 2019, another “power hour” was held at the conference titled “Immunology of Fungal Infections” [56]. Some challenges related to gender inequalities in science and actions to bridge the gap identified in these empowerment sessions were briefly summarized [55,56] in Table 1 in seven different groups.

Perhaps some less obvious challenges should be highlighted, such as the differential consideration of comments in scientific discussions and the differential distribution of tasks in research groups according to gender. Given the potential gaps in the biographies of women researchers, employers need to reflect on and learn why these gaps occurred. Nominating high-profile women scientists and researchers for awards is far more important than it might seem at first glance. A publicly accessible resource, such as a personal page in Wikipedia, can only be created for individuals who have received a significant award in a particular field. We need to imprint our female scientific ancestors from the analog era into digital memory. For undergraduate students to fully benefit from their undergraduate research experiences, undergraduate research opportunities should include a balanced representation of female mentors [57].

Most of these challenges have already been recognized by other researchers in science, technology, engineering, and mathematics, but we need to continue to talk and write about gender inequities in science; changes are going in the right direction, but perhaps not fast enough. Among other measures, organized retreats dedicated exclusively to writing scientific papers, in a quiet place away from work and home, could increase the number of required publications. Guidance included in gender-equality plans to keep senior women in science should be introduced.

### 3.4. What Are the Research Interests Identified in the Central Resource of Women Researchers of Filamentous Fungi and Oomycetes?

The underrepresentation of women among invited speakers is often attributed to a lack of appropriately qualified women. Therefore, in April 2016, the filamentous fungi and oomycetes research community produced a document entitled “Women Researchers in Fungi & Oomycetes” (WRIFFO) to serve as a central resource for identifying potential female speakers [37]. The goal of this resource is to improve the gender balance among speakers at conferences, which can be achieved through balanced speaker-appointment panels. The focus of this key resource is on positions at universities or equivalent institutions to provide critical support to women researchers (grade B and C) who still struggle to obtain independent research positions, particularly through invitations to scientific conference committees, scientific presentations, and other appointments. This comprehensive spreadsheet is quite simple, consisting of seven columns that include the researcher’s name, institution and country code, and geographic region. Researchers describe their research area in two ways: by two subfields selected from the drop-down menu and by keywords that can be freely chosen. There is also the possibility to indicate the research group website and the corresponding career stages [37].

**Figure 2:**
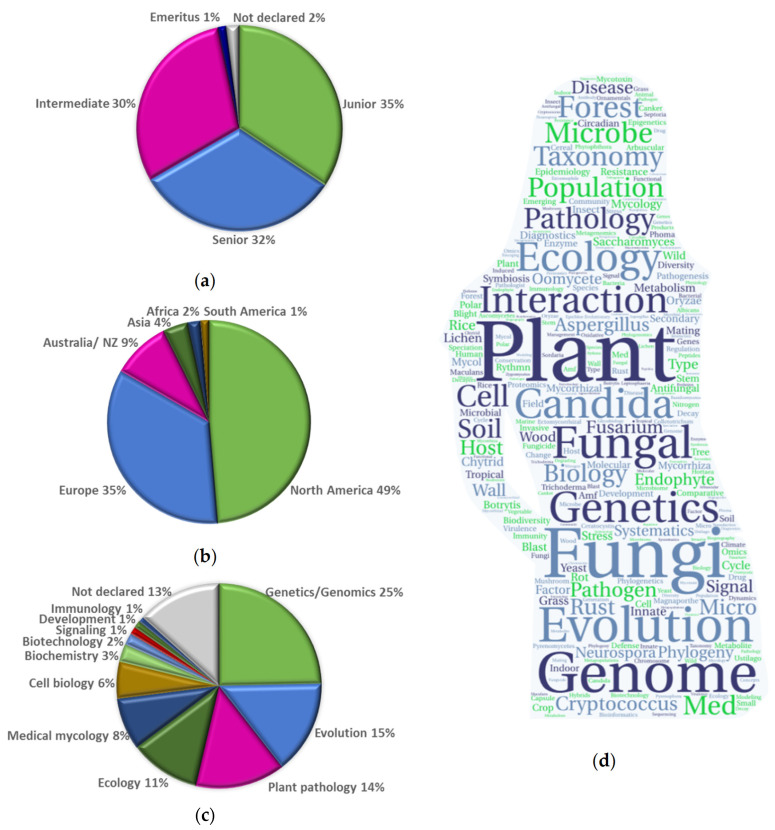
Women researchers voluntarily described in the central resource table of Women Researchers in Fungi & Oomycetes [37]. (**a**) Status, (**b**) region, (**c**) descriptors of fungal research area, and (**d**) word cloud of keywords used to describe research. Analysis of the 172 keywords inscribed at least twice or more (Table S9) using the online tool [38].

The corresponding career stages of the three hundred women researchers who voluntarily completed the table were 35% as assistant professor or in a similar position (Grade C), 32% as a full-time research position representing a professorship or equivalent (Grade A), and 30% in a mid-level position (Grade B) (Figure 2a, Table S6). Emeritus status was reported by 1% of researchers, and 2% did not provide information on their status. The geographic location of their laboratories was predominantly in North America (49%) and Europe (35%) (Figure 2a, Table S7). Only 16% of researchers were from other parts of the world—Australia and New Zealand 9%, Asia 4%, Africa 2%, and South America 1%.

From the descriptors used by researchers to describe the fungal research subfield, it can be concluded that the greatest interest was in genetics and genomics (25%), followed by evolution (15%), plant pathology (14%), ecology (11%), medical mycology (8%), cell biology (6%), biochemistry (3%), biotechnology (2%), development, immunology and signaling (1%). Only the descriptor taxonomy was not used, which may be because researchers in the fungal taxonomic society are not aware of this central resource database (Figure 2c, Table S8). The fungal research area was not indicated by 13% of the researchers.

Among the freely chosen keywords to indicate the research area, there were a total of four hundred different individual terms, which were evaluated using the online tool Tagul [38]. Up to 57% of the keywords for research area were unique, likely indicating that the women researchers were engaged in the full range of fungal research. Keywords that were used twice or more (172) are shown in Figure 2d (Table S9); the top 10% (forty keywords) were used between eight and sixty times. These freely chosen keywords may indicate that women scientists are more likely to focus on research areas such as fungi interacting with plants because they threaten our food or accelerate plant food production (mycorrhizae) and diagnosing pathogens because they cause disease in humans and animals. The fungal pathogens *Candida*, *Cryptococcus*, *Aspergillus*, and *Fusarium* are the most studied taxa, with *Saccharomyces* and *Neurospora* serving as models.

The major drawbacks of the data in this table are that they were voluntarily completed by researchers, or in some cases even completed for researchers, and that they do not include comparative data. Considering that there may be some areas of fungal research that are more likely to involve women scientists, it is important that editors and reviewers are not gender-biased in recognizing the importance of certain research in submitted manuscripts. Therefore, it is critical for fungal research that journals follow the Sex and Gender Equality in Research guidelines. A similar central source of information could be compiled for many other research areas.

### 3.5. Is There Progress in Reducing the Gender Gap at Fungal Genetics Conferences?

In the history of the five Gordon Research Conferences titled “Immunology of Fungal Infections” from 2011 to 2019, there has been a steady trend toward gender parity in the distribution of speakers. The proportion of women chairing sessions and organizing meetings nearly reached parity in 2019 [56]. Because most of the women researchers listed in the WRIFFO table (84%) were from North America and Europe (Figure 2a, Table S7), and a quarter of them reported genetics and genomics as their research area, progress in narrowing the gender gap was tracked at U.S. and European conferences on fungal genetics. The biennial conferences the Fungal Genetics Conference and the European Conference on Fungal Genetics, were selected for analysis [39,40,41,42]. Two of these conferences were held in Asilomar and Paris before 2016, when a central WRIFFO resource was established [15]. Two of the most recent conferences of the same type were held in Rome and Asilomar after 2019, when the so-called “power hours” were held as part of the Gordon Research Conferences [55,56].

**Figure 3:**
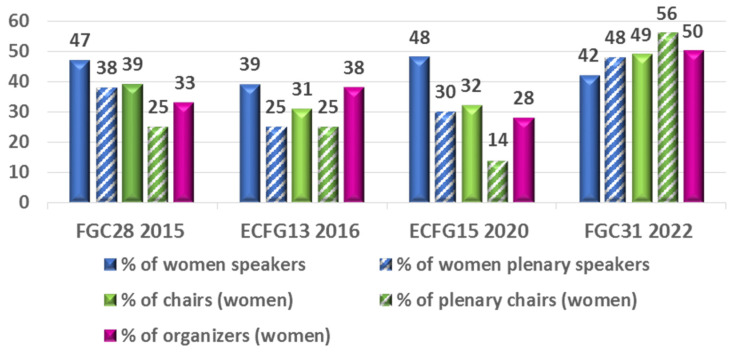
Breakdown of total speakers over time. Gender breakdown of all plenary and selected speakers for two alternating FGC and ECFG meetings is shown as percentage of women (Table S10). The conferences were chosen to take place before and after establishment of a central resource “Women researchers in fungi & oomycetes” in 2016 [37] and so called “power hours” were introduced at Gordon research conferences in 2017, 2018 and 2019 [55,56]. The breakdown of session chairs selected by the organizers at each meeting and gender breakdown of meeting organizers for each meeting is also shown as percentage of women. FGC28, The 28th Fungal Genetics Conference at Asilomar, 17–22 March 2015 [39]; ECFG13, The 13th European Conference on Fungal Genetics Paris, France, 3–6 April 2016 [40]; ECFG15; The 15th European Conference on Fungal Genetics Rome, Italy, 17–20 February 2020 [41]; and FGC31, The 31st Fungal Genetics Conference at Asilomar, 15–20 March 2022 [42].

Based on the announced names from the abstracts or program books, an (almost) balanced proportion of women speakers between 39% and 48% could be identified for all conferences studied (Figure 3, Table S10) [23,24,25,26]. Similar to the last, generally corresponding authorship in publications relative to overall authorship (Figure 1c), an un-derrepresented proportion of women speakers was observed in the more valuable an longer plenary presentations at FGC28, ECFG13, and ECFG15 in 2015, 2016, and 2020 (38%, 25%, and 30%, respectively) (Figure 3, Table S10). The proportion of women chairing sessions at FGC28, ECFG13, and ECFG15 in 2015, 2016, and 2020 ranged from an underrepresented 31% to 39%, with an additional significant decrease in chairing plenary sessions (14% to 25%) (Figure 3, Table S10). At FGC28, ECFG13, and ECFG15, held in 2015, 2016, and 2020, respectively, the underrepresented proportion of women scientists on committees ranged from 28% to 38% (Figure 3, Table S10).

However, the most recent conference, FGC31, held in 2022, showed a completely different picture. Although the overall proportion of women speakers was balanced (42%), it was even higher (48%) for the more important plenary presentations, reaching near parity among speakers (Figure 3, Table S10). The same positive trend was observed in the chairing of plenary sessions, where even more than half of the scientists were women (56%) compared to near parity of overall chair (49%) (Figure 3, Table S10). There was parity in committee membership by gender (Figure 3, Table S10). Unlike the FGC28, ECFG13, and ECFG15 conferences, the format of the most recent FGC31 conference was a hybrid, and included online speakers and participants, which may have affected the gender balance.

The balanced proportion of women researchers in overall speakers, (near) parity in plenary speakers, (near) parity of women researchers in overall chairs, balanced proportion in chairing plenary sessions in favor of women scientists, and parity in committee membership by gender observed at the recent Fungal Genetics Conference is very encouraging and gives hope that the gender gap can be closed. An increased number of fungal conferences rated by gender would further bolster confidence in the positive trends observed.

## Figures and Tables

**Table 1:** The challenges of gender inequalities in science and actions to bridge the gap.

Challenges of Gender Inequalities in Science	Actions to Bridge the Gap
(1) Awareness	✓ Write about gender gap in science; ✓ Promote fair peer-review; ✓ Nominate for awards.
(2) Unconscious sexual bias	✓ Recommendations; ✓ Considerations of comments; ✓ Assignment of tasks; ✓ Representation on committees.
(3) Mentoring	✓ Appoint women mentors for early career scientists, especially for women.
(4) Building and maintaining networks	✓ Retreats dedicated to writing scientific publications;
(5) Work–life balance	✓ Working-hours flexibility; ✓ Dare to say NO; ✓ Consider gaps in CVs.
(6) Pay bias	✓ Know your worth; ✓ Self-promote; ✓ Negotiate for salary in advance; ✓ Apply gender-blind hiring practices; ✓ Raise awareness about employment of minority applicants.
(7) Sexual harassment	✓ Know institutional procedures; ✓ Affirmation of code of conduct and role without harassment; ✓ Encourage to speak up when they feel it is necessary.

Summarized after [55,56].

## Supplementary Materials

The following supporting information can be downloaded at: https://www.mdpi.com/article/10.3390/jof8070675/s1, **Table S1**: The mycological journals; Table S2: Proportion of women authors publishing by research discipline; Table S3: Proportion of women researchers publishing by author position in microbiology; Table S4: Proportion of women researchers publishing by author position in mycology; Table S5: Research of fungal aegerolysins as published in scientific journals and disciplines; Table S6: Status of women researchers; Table S7: Region of women researchers; Table S8: Descriptors of fungal research area by women researchers; Table S9: Keywords women researchers use at least twice to describe their fungal research area; Table S10: Gender breakdown of plenary and selected speakers, session chairs, and meeting organizers for two alternating meetings FGC and ECFG; References [58,59,60,61,62,63,64,65,66,67,68,69,70,71,72,73,74,75,76,77,78,79,80,81,82,83,84,85,86,87,88,89,90] are cited in the supplementary materials.
