# Supplementary material for: Towards a Fungal Science That Is Independent of Researchers’ Gender
Source: J Fungi (Basel). 2022 Jun 28;8(7):675. doi: 10.3390/jof8070675 (PMC9321353; doi:10.3390/jof8070675)
Supplement: Supplementary file 1 [file jof-08-00675-s001.zip › 2.jof-1746342-supplementary.pdf]

Supplementary Materials

Table S1. The mycological journals

Title of the serial publication	Discipline	Overall 2016	Overall 2022	First 2016	First 2022	Last 2016	Last 2022
Eukaryotic Cell	Cell Biology	43%	44.4%	50%	50%	27%	28%
FEMS Yeast Research	Microbiology	44.8%	45%	50.0%	50%	37.3%	34.7
Fungal Biology	Microbiology	46.4%	45%	53.4%	50%	39.7%	36%
Fungal Genetics and Biology	Genetics	43%	41.5%	49.2%	49.7%	27.3%	29.9%
Medical Mycology	Microbiology	47.2%	48.6%	49.1%	49.8%	33.3%	32%
Mycologia	Microbiology	33.8%	33.5%	40.4%	41.6%	32.2%	36%
Mycopathologia	Microbiology	50%	49.7%	49.8%	50%	38.4%	39.2%
Mycorrhiza	Botany	48.3%	46.1%				
Mycoses	Microbiology	48.1%	44.4%	50%	50%	28.5%	19.9%
Revista Iberoamericana de Micologia	Microbiology	49.7%	50%				
Yeast	Microbiology	41.2%	45.2%	49.6%	49.9%	33%	38.2%
Nature	Multidisciplinary	31.4%	34.9%	28%	30.1%	18.3%	20.8%
Science	Multidisciplinary	30%	30.7%	31.2%	31.2%	21.9%	23.4%

Mycology journals according to the Journal Citation Reports for 2016 [1]. Proportion of women authors according to the discipline search in the web app The Gender Gap in Academic Publishing [2]. Additional journal titled Mycological Research was discontinued in 2011; green, 50% or more; blue 40%-60%.

Table S2. Proportion of women authors publishing by research discipline

Discipline	2016	Prediction for 2022
Immunology	44.2%	46%
Microbiology	43.1%	44.9%
Genetics	41.5%	43.1%
Toxicology	41.5%	44.8
Cell Biology	40.9%	42.9%
Botany	38.5%	41%
Medicine	38.5%	40.9%
Biotechnology	37.9%	41.9%
Biology	37.3%	39.4%
Multidisciplinary	37.1%	40.8%
Biochemistry	36.4%	39.1%
Biophysics	33.4%	33%
Zoology	31%	28.9%

Proportion as percentage of women authors according to the discipline search in the web app The Gender Gap in Academic Publishing [2].

Table S3. Proportion of women researchers publishing by author position in microbiology

Microbiology	2016	Prediction for 2022
First	51.5%	50%
Single	38.5%	44.1%
Overall	43.1%	44.9%
Last	32.2%	36.4%

Proportion as percentage of women researchers according to the author position in microbiology the web app The Gender Gap in Academic Publishing [2].

Table S4. Proportion of women researchers publishing by author position in mycology

Mycology	2016	Prediction for 2022
First	49.1%*	49%*
Overall	45.1%	45%
Last	33%*	32.7%*

Proportion as percentage of women researchers according to the author position in microbiology to the web app The Gender Gap in Academic Publishing [2] calculated from the eleven mycological journals (Journal Citation Reports 2016 [1]); *, data for nine mycological journals.

Table S5. Research of fungal aegerolysins as published in scientific journals and disciplines

Journal	Discipline	Reference
BMC Microbiology	Microbiology	[3]
FEMS Microbiology Letters	Microbiology	[4]
Microbiology	Microbiology	[5]
Molecular Microbiology	Microbiology	[6]
Mycological Research	Microbiology	[7]
Medical Mycology	Microbiology/ Mycology	[8,9]
Mycopathologia	Microbiology/ Mycology	[10]
Biochemistry	Biochemistry	[11]
Biochimie	Biochemistry	[12,13]
Chemistry and Physics of Lipids	Biochemistry	[14]
FEBS Letters	Biochemistry	[15]
Protein science	Biochemistry	[16]
Sub-Cellular Biochemistry	Biochemistry	[17]
Biochimica et Biophysica Acta	Biophysics	[18,19,20]
Applied Microbiology and Biotechnology	Biotechnology	[21,22]
Seminars in Cell & Developmental Biology	Cell biology	[23]
The FASEB Journal	Cell biology	[24]
International Journal of Medical Microbiology	Medicine	[25]
International Journal of Medical Sciences	Medicine	[26]
Molecular Genetics and Genomics	Genetics	[27]
Developmental and Comparative Immunology	Immunology	[28]
PloS One	Multidisciplinary	[29]
Toxicon	Toxicology	[30]
Journal of Invertebrate Pathology	Zoology	[31]
International Journal of Medicinal Mushrooms	/	[32]
Microorganisms	/	[33]
Mycological Progress	/	[34]
Scientific Reports	/	[35,36]
Toxins	/	[37,38,39]

The research of fungal aegerolysins according to Scopus title-abstract-keywords search (TITLE-ABS-KEY (aegerolysin*) AND TITLE-ABS-KEY (fung*)) [40]. Papers found (37) were according to journals assigned to appropriate disciplines by the web app The Gender Gap in Academic Publishing [2].

Table S6. Status of women researchers

Status	Number
Emeritus	4
Intermediate	90
Senior	97
Junior	103
Not declared	6

Table S7. Region of women researchers

Region	Number
Africa	6
Asia	12
Australia/ NZ	28
Europe	104
North America	146
South America	4

Table S8. Descriptors of fungal research area by women researchers

Descriptors	Number of descriptor repetition
Biochemistry	17
Biotechnology	11
Cell biology	35
Development	7
Ecology	67
Evolution	89
Genetics/Genomics	148
Immunology	5
Medical mycology	49
Plant pathology	85
Signaling	7
Taxonomy	0
Not declared	80

Table S9. Keywords women researchers use at least twice to describe their fungal research area

Keywords	[1] Number of repetitions
**plant**	**60**
**genome**	**52**
**interaction**	**41**
**evolution**	**38**
***Candida*, fungi, genetics**	**35**
**fungal**	**34**
**ecology**	**33**
**pathology**	**32**
**population**	**27**
**taxonomy**	**23**
**microbe**	**22**
**pathogen**	**20**
**forest**	**19**
**biology**	**17**
Cryptococcus	**16**
***Aspergillus*, cell**	**15**
**medicine**	**14**
**phylogeny**	**13**
**endophyte, *Fusarium,* oomycetes, systematics**	**12**
**Disease, *Neurospora*, *Saccharomyces***	**11**
**diagnostics, micro**	**10**
**development, metabolism, mycology, mycorrhizal, rust, signal**	**9**
**epidemiology, host, mycorrhiza, soil**	**8**
antifungal, biodiversity, *Magnaporthe*, secondary, symbiosis	7
phylogenetics, resistance	6
Botrytis, chytrid, comparative, enzyme, molecular, tropical, wall,	5
arbuscular, biotechnology, circadian, community, diversity, ectomycorrhizal, immunity, innate, lichen, mycotoxin, *Phytophthora*, *Trichoderma*, wood	4
Ascomycetes, immunology, *oryzae*, rhythm, factor, epigenetics, metabolite, mating, virulence, Pyrenomycetes, metagenomics, type, emerging, invasive, regulation, grass, fungicide, proteomics, speciation, pathologist, stress, med(ical), mycology, mushroom, species, *Colletotrichum*	3
albicans, AMF, animal, antibody, Bacteria, bacterial, Basidiomycetes, biogeography, bioinformatics, blast, blight, canker, capsule, *Ceratocystis*, cereal, change, chromosome, climate, concepts, conservation, crop, cycle, decay, decayers, defense, degrading, drug, dynamics, *Epichloe*, evolutionary, extremophile, field, functional, generation, genes, glycobiology, *Hortaea*, human, hybrids, indoor, induced, insect, *Leptosphaeria*, lignocellulolytic, *maculans*, management, marine, metapopulations, microbiome, modeling, Mucoromycotina, nitrogen, omics, ornamentals, oxidative, pathogenomics, peptides, *Phoma*, phylogenomics, physiology, polar, products, *Pyrenophora*, rice, rot, *Septoria*, sequencing, small, *Sordaria*, stem, transduction, tree, *Ustilago*, vegetable, wild, yeast, Zygomycetes	2

The top 10% or top 40 keywords used by Women Researchers in Fungi & Oomycetes (WRIFFO) [41] are listed in bold. Analysis using the online tool Tagul [42]. [1] Number of keyword repetitions in the WRIFFO table[41]. AMF, arbuscular mycorrhiza fungus.

Table S10. Gender breakdown of plenary and selected speakers, session chairs, and meeting organizers for two alternating meetings FGC and ECFG

Conference	Number of speakers women + men	Number of plenary speakers women + men	Number of chairs women + men	Number of chairs in plenary sessions women + men	Number of organizers women + men
**FGC28 2015**	101 + 111 (2?)	8 + 13	22 + 34	1 + 3	5 + 10
**ECFG13 2016**	36 + 56	5 + 15	9 + 20	3 + 9	14 + 23
**ECFG15 2020**	53 + 57	6 + 14	10 + 21	2 + 5	15 + 39
**FGC31 2022**	102 + 119 (3?)	10 + 11	32 + 33	5 + 4	8 + 8

FGC28, The 28th Fungal Genetics Conference at Asilomar, March 17-22, 2015 [43]; ECFG13, The 13th European Conference on Fungal Genetics Paris, France, April 3-6, 2016 [44]; ECFG15; The 15th European Conference on Fungal Genetics Rome, Italy, February 17-20, 2020 [45]; and FGC31, The 31st Fungal Genetics Conference at Asilomar, March 15 - 20, 2022 [46]; ?, unknown gender of the speaker.

References

1. Clarivate analytics Available online: https://clarivate.com/ (accessed on Nov 30, 2021).
2. Holman, L.; Lloyd, E. Gender gap in academic publishing Available online: https://lukeholman.github.io/genderGap/ (accessed on Nov 30, 2021).
3. Pires, A.B.L.; Gramacho, K.P.; Silva, D.C.; Góes-Neto, A.; Silva, M.M.; Muniz-Sobrinho, J.S.; Porto, R.F.; Villela-Dias, C.; Brendel, M.; Cascardo, J.C.M.; et al. Early development of Moniliophthora perniciosa basidiomata and developmentally regulated genes. *BMC Microbiol.* **2009**, *9*, 158, doi:10.1186/1471-2180-9-158.
4. Miklavič, Š.; Kogovšek, P.; Hodnik, V.; Korošec, J.; Kladnik, A.; Anderluh, G.; Gutierrez-Aguirre, I.; Maček, P.; Butala, M. The Pseudomonas aeruginosa RhlR-controlled aegerolysin RahU is a low-affinity rhamnolipid-binding protein. *FEMS Microbiol. Lett.* **2015**, *362*, fnv069–fnv069, doi:10.1093/femsle/fnv069.
5. Rao, J.; DiGiandomenico, A.; Unger, J.; Bao, Y.; Polanowska-Grabowska, R.K.; Goldberg, J.B. A novel oxidized low-density lipoprotein-binding protein from Pseudomonas aeruginosa. *Microbiology* **2008**, *154*, 654–665, doi:10.1099/mic.0.2007/011429-0.
6. Novak, M.; Čepin, U.; Hodnik, V.; Narat, M.; Jamnik, M.; Kraševec, N.; Sepčić, K.; Anderluh, G. Functional studies of aegerolysin and MACPF-like proteins in Aspergillus niger. *Mol. Microbiol.* **2019**, *112*, 1253–1269, doi:10.1111/mmi.14360.
7. Vidic, I.; Berne, S.; Drobne, D.; Maček, P.; Frangež, R.; Turk, T.; Štrus, J.; Sepčić, K. Temporal and spatial expression of ostreolysin during development of the oyster mushroom (Pleurotus ostreatus). *Mycol. Res.* **2005**, *109*, 377–382, doi:10.1017/S0953756204002187.
8. Maličev, E.; Chowdhury, H.H.; Maček, P.; Sepčić, K. Effect of ostreolysin, an Asp-hemolysin isoform, on human chondrocytes and osteoblasts, and possible role of Asp-hemolysin in pathogenesis. *Med. Mycol.* **2007**, *45*, 123–130, doi:10.1080/13693780601039615.
9. Nayak, A.P.; Green, B.J.; Beezhold, D.H. Fungal hemolysins. *Med. Mycol.* **2013**, *51*, 1–16, doi:10.3109/13693786.2012.698025.
10. Nayak, A.P.; Blachere, F.M.; Hettick, J.M.; Lukomski, S.; Schmechel, D.; Beezhold, D.H. Characterization of recombinant terrelysin, a hemolysin of Aspergillus terreus. *Mycopathologia* **2011**, *171*, 23–34, doi:10.1007/s11046-010-9343-0.
11. Berne, S.; Sepčić, K.; Anderluh, G.; Turk, T.; Maček, P.; Poklar Ulrih, N.N.; Sepcić, K.; Anderluh, G.; Turk, T.; Macek, P.; et al. Effect of pH on the pore forming activity and conformational stability of ostreolysin, a lipid raft-binding protein from the edible mushroom Pleurotus ostreatus. *Biochemistry* **2005**, *44*, 11137–47, doi:10.1021/bi051013y.
12. Hullin-Matsuda, F.; Makino, A.; Murate, M.; Kobayashi, T. Probing phosphoethanolamine-containing lipids in membranes with duramycin/cinnamycin and aegerolysin proteins. *Biochimie* **2016**, *130*, 81–90, doi:10.1016/j.biochi.2016.09.020.
13. Ota, K.; Leonardi, A.; Mikelj, M.; Skočaj, M.; Wohlschlager, T.; Künzler, M.; Aebi, M.; Narat, M.; Križaj, I.; Anderluh, G.; et al. Membrane cholesterol and sphingomyelin, and ostreolysin A are obligatory for pore-formation by a MACPF/CDC-like pore-forming protein, pleurotolysin B. *Biochimie* **2013**, *95*, 1855–1864, doi:10.1016/j.biochi.2013.06.012.
14. Hullin-Matsuda, F.; Murate, M.; Kobayashi, T. Protein probes to visualize sphingomyelin and ceramide

phosphoethanolamine. *Chem. Phys. Lipids* **2018**, *216*, 132–141, doi:10.1016/j.chemphyslip.2018.09.002.
15. Sepčić, K.; Berne, S.; Rebolj, K.; Batista, U.; Plemenitaš, A.; Šentjurc, M.; Maček, P. Ostreolysin, a pore-forming protein from the oyster mushroom, interacts specifically with membrane cholesterol-rich lipid domains. *FEBS Lett.* **2004**, *575*, 81–85, doi:10.1016/j.febslet.2004.07.093.
16. Berne, S.; Lah, L.; Sepčić, K. Aegerolysins: structure, function, and putative biological role. *Protein Sci.* **2009**, *18*, 694–706, doi:10.1002/pro.85.
17. Ota, K.; Butala, M.; Viero, G.; Dalla Serra, M.; Sepčić, K.; Maček, P. Fungal MACPF-Like Proteins and Aegerolysins: Bi-component Pore-Forming Proteins? In; 2014; pp. 271–291.
18. Skočaj, M.; Yu, Y.; Grundner, M.; Resnik, N.; Bedina Zavec, A.; Leonardi, A.; Križaj, I.; Guella, G.; Maček, P.; Kreft, M.E.; et al. Characterisation of plasmalemmal shedding of vesicles induced by the cholesterol/sphingomyelin binding protein, ostreolysin A-mCherry. *Biochim. Biophys. Acta - Biomembr.* **2016**, *1858*, 2882–2893, doi:10.1016/j.bbamem.2016.08.015.
19. Novak, M.; Krpan, T.; Panevska, A.; Shewell, L.K.; Day, C.J.; Jennings, M.P.; Guella, G.; Sepčić, K. Binding specificity of ostreolysin A6 towards Sf9 insect cell lipids. *Biochim. Biophys. Acta - Biomembr.* **2020**, *1862*, 183307, doi:10.1016/j.bbamem.2020.183307.
20. Berne, S.; Križaj, I.; Pohleven, F.; Turk, T.; Maček, P.; Sepčić, K. Pleurotus and Agrocybe hemolysins, new proteins hypothetically involved in fungal fruiting. *Biochim. Biophys. Acta* **2002**, *1570*, 153–159, doi:10.1016/S0304-4165(02)00190-3.
21. Ngai, P.H.K.; Ng, T.B. A hemolysin from the mushroom Pleurotus eryngii. *Appl. Microbiol. Biotechnol.* **2006**, *72*, 1185–1191, doi:10.1007/s00253-006-0406-6.
22. Novak, M.; Kraševec, N.; Skočaj, M.; Maček, P.; Anderluh, G.; Sepčić, K. Fungal aegerolysin-like proteins: distribution, activities, and applications. *Appl. Microbiol. Biotechnol.* **2015**, *99*, 601–610, doi:10.1007/s00253-014-6239-9.
23. Butala, M.; Novak, M.; Kraševec, N.; Skočaj, M.; Veranič, P.; Maček, P.; Sepčić, K. Aegerolysins: Lipid-binding proteins with versatile functions. *Semin. Cell Dev. Biol.* **2017**, *72*, 142–151, doi:10.1016/j.semcdb.2017.05.002.
24. Bhat, H.B.; Ishitsuka, R.; Inaba, T.; Murate, M.; Abe, M.; Makino, A.; Kohyama-Koganeya, A.; Nagao, K.; Kurahashi, A.; Kishimoto, T.; et al. Evaluation of aegerolysins as novel tools to detect and visualize ceramide phosphoethanolamine, a major sphingolipid in invertebrates. *FASEB J.* **2015**, *29*, 3920–3934, doi:10.1096/fj.15-272112.
25. Wartenberg, D.; Lapp, K.; Jacobsen, I.D.; Dahse, H.-M.; Kniemeyer, O.; Heinekamp, T.; Brakhage, A.A. Secretome analysis of Aspergillus fumigatus reveals Asp-hemolysin as a major secreted protein. *Int. J. Med. Microbiol.* **2011**, *301*, 602–611, doi:10.1016/j.ijmm.2011.04.016.
26. Yap, H.-Y.Y.; Fung, S.-Y.; Ng, S.-T.; Tan, C.-S.; Tan, N.-H. Genome-based Proteomic Analysis of Lignosus rhinocerotis (Cooke) Ryvarden Sclerotium. *Int. J. Med. Sci.* **2015**, *12*, 23–31, doi:10.7150/ijms.10019.
27. Dubey, M.; Jensen, D.F.; Karlsson, M. Functional characterization of the AGL1 aegerolysin in the mycoparasitic fungus Trichoderma atroviride reveals a role in conidiation and antagonism. *Mol. Genet. Genomics* **2021**, *296*, 131–140, doi:10.1007/s00438-020-01732-3.
28. Zhang, S.; Clark, K.D.; Strand, M.R. The protein P23 identifies capsule-forming plasmatocytes in the moth Pseudoplusia includens. *Dev. Comp. Immunol.* **2011**, *35*, 501–510, doi:10.1016/j.dci.2010.12.006.
29. Kelker, M.S.; Berry, C.; Evans, S.L.; Pai, R.; McCaskill, D.G.; Wang, N.X.; Russell, J.C.; Baker, M.D.; Yang, C.; Pflugrath, J.W.; et al. Structural and Biophysical Characterization of Bacillus thuringiensis Insecticidal Proteins Cry34Ab1 and Cry35Ab1. *PLoS One* **2014**, *9*, e112555, doi:10.1371/journal.pone.0112555.
30. Juntes, P.; Rebolj, K.; Sepčić, K.; Maček, P.; Cecilija Žužek, M.; Cestnik, V.; Frangež, R. Ostreolysin induces sustained contraction of porcine coronary arteries and endothelial dysfunction in middle- and large-sized vessels. *Toxicon* **2009**, *54*, 784–792, doi:10.1016/j.toxicon.2009.06.005.
31. Panevska, A.; Skočaj, M.; Modic, Š.; Razinger, J.; Sepčić, K. Aegerolysins from the fungal genus Pleurotus – Bioinsecticidal proteins with multiple potential applications. *J. Invertebr. Pathol.* **2021**, *186*, 107474, doi:10.1016/j.jip.2020.107474.
32. Rebolj, K.; Sepcic, K. Ostreolysin, a Cytolytic Protein from Culinary-Medicinal Oyster Mushroom Pleurotus ostreatus (Jacq.: Fr.) P. Kumm. (Agaricomycetideae), and Its Potential Use in Medicine and Biotechnology. *Int. J. Med. Mushrooms* **2008**, *10*, 293–302, doi:10.1615/IntJMedMushr.v10.i4.10.
33. Kraševec, N.; Novak, M.; Barat, S.; Skočaj, M.; Sepčić, K.; Anderluh, G. Unconventional Secretion of Nigerolysins A from Aspergillus Species. *Microorganisms* **2020**, *8*, 1973, doi:10.3390/microorganisms8121973.
34. Roberts, R.G.; Bischoff, J.F.; Reymond, S.T. Differential gene expression in Alternaria gaisen exposed to dark and light. *Mycol. Prog.* **2011**, *11*, 373–382, doi:10.1007/s11557-011-0752-3.
35. Kočar, E.; Lenarčič, T.; Hodnik, V.; Panevska, A.; Huang, Y.; Bajc, G.; Kostanjšek, R.; Naren, A.P.; Maček, P.; Anderluh, G.; et al. Crystal structure of RahU, an aegerolysin protein from the human pathogen Pseudomonas aeruginosa, and its interaction with membrane ceramide phosphorylethanolamine. *Sci. Rep.* **2021**, *11*, 6572, doi:10.1038/s41598-021-85956-2.
36. Panevska, A.; Hodnik, V.; Skočaj, M.; Novak, M.; Modic, Š.; Pavlic, I.; Podržaj, S.; Zarić, M.; Resnik, N.; Maček, P.; et al. Pore-forming protein complexes from Pleurotus mushrooms kill western corn rootworm and Colorado potato beetle through targeting membrane ceramide phosphoethanolamine. *Sci. Rep.* **2019**, *9*, 5073, doi:10.1038/s41598-019-

41450-4.
37. Milijaš Jotić, M.; Panevska, A.; Iacovache, I.; Kostanjšek, R.; Mravinec, M.; Skočaj, M.; Zuber, B.; Pavšič, A.; Razinger, J.; Modic, Š.; et al. Dissecting Out the Molecular Mechanism of Insecticidal Activity of Ostreolysin A6/Pleurotolysin B Complexes on Western Corn Rootworm. *Toxins (Basel).* **2021**, *13*, 455, doi:10.3390/toxins13070455.
38. Kraševec, N.; Panevska, A.; Lemež, Š.; Razinger, J.; Sepčić, K.; Anderluh, G.; Podobnik, M. Lipid-Binding Aegerolysin from Biocontrol Fungus Beauveria bassiana. *Toxins (Basel).* **2021**, *13*, 820, doi:10.3390/toxins13110820.
39. Panevska, A.; Glavan, G.; Jemec Kokalj, A.; Kukuljan, V.; Trobec, T.; Žužek, M.C.; Vrecl, M.; Drobne, D.; Frangež, R.; Sepčić, K. Effects of Bioinsecticidal Aegerolysin-Based Cytolytic Complexes on Non-Target Organisms. *Toxins (Basel).* **2021**, *13*, 457, doi:10.3390/toxins13070457.
40. Scopus Search Available online: https://www.scopus.com/search/form.uri?display=basic (accessed on May 9, 2022).
41. Momany, M.; Jason, S. Women researchers in fungi & oomycetes Available online: http://fungalgenomes.org/blog/wrifo/ (accessed on Jan 17, 2020).
42. Tagul word cloud art creator Available online: https://wordart.com/ (accessed on Mar 13, 2020).
43. The 28th Fungal Genetics Conference at Asilomar, March 17-22, 2015, Program book Available online: https://www2.genetics-gsa.org/fungal/2015/index.shtml (accessed on Apr 20, 2022).
44. The 13th European Conference on Fungal Genetics Paris, France, April 3-6, 2016, Abstracts Book Available online: https://hal.inrae.fr/hal-02792296/file/2016-1_1.pdf (accessed on Apr 20, 2022).
45. The 15th European Conference on Fungal Genetics Rome, Italy, February 17-20, 2020, Conference Abstracts Book Available online: https://www.ecfg15.org/ (accessed on Apr 22, 2022).
46. The 31st Fungal Genetics Conference at Asilomar, March 15 - 20, 2022, Program Book Available online: https://genetics-gsa.org/fungal-2022/program-and-abstract-books/ (accessed on Apr 20, 2022).
